# Current News and Opinions

**Published:** 1888-10

**Authors:** 


### Current News and Opinions.

### UNION MEETING.

The Fifth, Sixth, Seventh and Eighth District Dental Societies of the State of New York will unite in a joint convention at the Leland Hotel, Syracuse, Oct. 24th, 25th, and 26th.

The meeting will be called to order at 2 o'clock.

A cordial invitation is extended to the profession to be present.

An extensive programme has been arranged.

Prominent dentists from Chicago, Baltimore, Cincinnati, Philadelphia, New York, Boston, Toronto, Newark, Albany and other cities have consented to take part.

One-half day will be devoted to clinics, and demonstrations of improved methods of work and new appliances.

All the leading dental manufacturing companies have arranged to be present with a full line of their goods. They expect to outdo previous exhibitions in this section of the State.

Among the social features of the convention will be a banquet for the dentists and a reception for the ladies.

It is expected that dentists will bring their wives.

A ladies' committee has been appointed.

Syracuse. G. L. CURTIS, Chairman Business Committee.

---

The Fifth Annual Meeting of the Ohio State Dental Society (reorganized) will be held in Cincinnati, Oct. 16th, 17th and 18th, in Lincoln Club Hall, Garfield Place and Race St. A cordial invitation is extended to the members of the Profession to attend and bring their families.

Railroad rates on account of the Centennial Exposition are very low.

E. G. BETTY, Ex. Com.

PROGRAMME.

I. Obtunding Sensitive Dentistry. Levitt E. Custer, Dayton.
II. Pericementitis. F. S. Maxwell, Stubenville.
III. Machinery as Used in Dentistry. C. R. Butler, Cleveland.
IV. Implantation, Replantation and Transplantation. H. A. Smith, Cincinnati.
V. Examining the Teeth and Jaws of Skulls. E. G. Betty, Cincinnati.
VI. Dental Education. J. Taft, Cincinnati.
VII. Medical Education for Dentists. C. M. Wright, Cincinnati.
VIII. Volunteer Papers.

CLINICS.

Methods of Cleaning Teeth. Wm. Taft, Cincinnati.
Implantation. M. H. Fletcher, Cincinnati.
Operative Dentistry. D. W. Clancy, Cincinnati.

---

### CAMPHORATED CARBOLIC ACID.

In the *Correspondenzblatt für Schweitzer Aerzte*, No. 7, 1888, Dr. Theodore Schneider (Basle) recommends camphorated carbolic acid as an "elegant, reliable, and very convenient antiseptic preparation." As is well known, when one part of crystallized carbolic acid and three parts of powdered camphor are shaken up together in a test-tube, a colorless, limpid fluid is produced. This mixture does not possess either the characteristic odor or the rubefacient and caustic properties of carbolic acid, while the antiseptic power of the latter remains intact. When placed on the tongue, the compound causes but very slight burning sensations. It has no effect on polished steel.—*British Med. Journal.*

This preparation would form an elegant solution in which to keep nerve broaches.—ED.

---

### ACID CORROSIVE SUBLIMATE SOLUTION.

Dr. O. Bujwid (Warsaw) has used for the last two years an addition of 2 per cent. hydrochloric acid to 1 to 1000 solutions of bichloride of mercury. This solution is more certain than neutral and alkaline solutions of sublimate, which readily precipitate an albuminate of mercury. It is prepared by dissolving 5 grammes of sublimate in 10 grammes of hydrochloric acid under heat, and then mixing with 5 litres of water. The sulphates and carbonates present in water are decomposed by the acid, and are without action. Other surgeons in Warsaw have used the same solution for some time with good results.—Translated from *Centralblatt für Bacteriologie*, No. 3, 1888.

### THE MICROBE OF TETANUS.

The bacillary origin of tetanus is rapidly being placed on a sound basis. In some recent experiments with a certain bacillus, which is credited with this pathogenic power, forty-five guinea pigs, seventeen rabbits, two lambs and one sheep were inoculated with a cultivation, with the result that twenty-seven of the animals died of well marked tetanus, twelve suffered from tetanic symptoms, from which they recovered, and ten died from acute systemic infection without tetanic manifestations. Although the investigation bore on the pathology of idiopathic tetanus, it is highly probable that traumatic tetanus is due to the same cause.—*Med. Press and Circular.*

Dr. Bell Taylor writes in the *British Medical Journal:* M. Pasteur's treatment has already been followed by 136 deaths; the vast majority of the people who have visited him were in no sort of danger, and it does seem a pity to induce such patients to incur the terrible risks inseparable from the hypodermic injection of rabid matter. Dr. Lutaud, chief editor of the *Journal de Médicine de Paris*, says: M. Pasteur does not cure hydrophobia; he gives it, and perhaps he is right.

Dr. G. D. Sitherwood, Bloomington, Ill., uses lanoline as a menstruum, also glycerine. He prefers the following formula for eucalyptus:

℞ Oleum eucalypti ........................................ ♏x

Glycerine ........................................ ℥j

M. Sig.—Apply locally.

For an atiseptic and prophylactic tooth powder, he adds to an ordinary powder one-half its bulk of acidum boricine.

Ohio State Dental Society.—The fourth annual meeting of the Ohio State Dental Society will be held in Cincinnati, October 16, 17 and 18, 1888.

J. R. Callahan, Hillsboro, Ohio, Secretary     Jere. E. Robinson, President

Beginning with the session of 1888-89, the College of Physicians and Surgeons, New York, following the example of Jefferson Medical College, Philadelphia, will require a three year's course of study from its students.

The Medico-Chirurgical College, Philadelphia, also re-establishes its three years' course, which had been lowered last year to two years' actual attendance and one year with preceptor.

A New Medical College has been incorporated in Brooklyn, known as "The College of Physicians and Surgeons of St. Mary's Hospital of the City of Brooklyn."

The cost of the last International Medical Congress in Washington was over $54,000, much exceeding that of any one of the previous three.

Koller, the discoverer of cocaine, has removed from Vienna to New York.

